# Biosynthetic potential of the culturable foliar fungi associated with field-grown lettuce

**DOI:** 10.1007/s00253-025-13581-4

**Published:** 2025-09-09

**Authors:** Neda Arad, Joseph Spraker, Kayla Garcia, Duke Pauli, A. Elizabeth Arnold

**Affiliations:** 1 School of Plant Sciences, The University of Arizona, 1140 E South Campus Drive, Forbes 303, Tucson, AZ 85721 USA; 2 Ecosystem Genomics Graduate Interdisciplinary Program, The University of Arizona, 1140 E South Campus Drive, Forbes 303, Tucson, AZ USA; 3 Hexagon Bio, Menlo Park, CA 94025 USA; 4 Center for Agroecosystem Research in the Desert (ARID), The University of Arizona, 1140 E South Campus Drive, 303 Tucson, Tucson, AZ USA; 5 BIO5 Institute and Department of Ecology and Evolutionary Biology, The University of Arizona, 1140 E South Campus Drive, Forbes 303, Tucson, AZ 85721 USA

**Keywords:** Comparative genomics, Endophyte, Fungi, Lettuce, Secondary metabolites

## Abstract

**Abstract:**

Fungal endophytes and epiphytes associated with plant leaves can play important ecological roles through the production of specialized metabolites encoded by biosynthetic gene clusters (BGCs). However, their functional capacity, especially in crops like lettuce (*Lactuca sativa* L.), remains poorly understood. We sequenced the genomes of nine fungal isolates, representing *Fusarium* sp., *Fulvia* sp., *Alternaria alternata*, and *Alternaria postmessia*, from leaves of lettuce grown under field conditions in Arizona, USA. We used antibiotics and secondary metabolite analysis shell (antiSMASH) and the database for automated carbohydrate-active enzyme annotation (dbCAN3), to predict BGCs and carbohydrate-active enzymes (CAZymes) for each strain, and then compared them to conspecific strains from other environments and substrates. Foliar lettuce-associated fungi featured 39–95 BGCs per genome, with substantial overlap between isolates occurring in association with lettuce leaves vs. from other substrates. Species identity was a significant determinant of BGC count, while host type, isolation source, and lifestyle were not. Several BGCs, including those for alternariol and 1,3,6,8-Tetrahydroxynaphthalene (T4HN), showed 100% similarity to characterized minimum information about a biosynthetic gene cluster (MIBiG) clusters based on antiSMASH predictions. Although analysis by biosynthetic gene similarity clustering and prospecting engine (BiG-SCAPE) identified gene cluster families (GCFs) across the dataset, these reference-matching clusters were not always grouped, reflecting methodological differences in how the tools assess similarity. Comparative CAZyme analysis in a focal species (*Fulvia* sp.) revealed higher gene counts in a foliar lettuce-derived isolate than in tomato (*Solanum lycopersicum*)-associated strains, challenging assumptions about host chemical complexity. These results highlight the importance of phylogenetic context in shaping fungal functional potential and suggest that selection on microbial traits in edible leafy crops may be more subtle and species-specific than previously assumed.

**Key points:**

• *Lettuce-associated fungi feature diverse biosynthetic potential*

• *Phylogeny predicts fungal BGC content more strongly than ecological lifestyle*

• *Findings support genome-informed microbiome strategies for leafy crops*

## Introduction

Fungal endophytes, which colonize plant tissues without causing visible indications of disease, are important in plant health, development, resilience, and microbial community dynamics (de Bary 1866; Arnold et al. 2003; Rodriguez et al. 2009; Hardoim et al. 2015; Liu et al. 2020; Oleńska et al. 2020; Fadiji and Babalola 2020a). These fungi produce a variety of specialized metabolites, many of which are synthesized by biosynthetic gene clusters (BGCs), tightly linked sets of genes that collectively encode enzymes for secondary metabolite biosynthesis (Rokas et al. 2020). These metabolites include alkaloids, polyketides, terpenes, nonribosomal peptides, and other small molecules with roles in antimicrobial activity, stress tolerance, host colonization, and chemical signaling. In the context of plant–microbe interactions, BGC-encoded compounds can help fungi suppress plant defenses, compete with other organisms, or endure abiotic stressors such as drought or UV radiation, thereby influencing ecological success and adaptation (Lugtenberg et al. 2016; Fadiji and Babalola 2020b; Crits-Christoph et al. 2021). Among tools available today (e.g., Zhou et al. 2022), the antibiotics and secondary metabolite analysis shell pipeline (antiSMASH) allows for efficient prediction of fungal BGCs from genome sequences (Blin et al. 2021), supporting comparative studies at various scales (Knapp et al. 2018; Jing et al. 2022).

Recently, genome sequencing has revealed that the number and types of BGCs can vary dramatically even among closely related fungal species, with habitat or substrate often playing a significant role in this variability (Knapp et al. 2018; Rokas et al. 2020; Jing et al. 2022; Allen et al. 2024). For instance, endophytic fungi that are transmitted horizontally often possess numerous BGCs related to plant interaction or defense suppression (Slot and Rokas 2011; Scott et al. 2023). In contrast, some obligate fungi exhibit signs of BGC reduction or pseudogenization, potentially due to relaxed selection pressures or niche specialization (Jing et al. 2022). The interplay of ecological filtering and phylogenetic constraints in fungi has been explored in several studies, often with a focus on root-associated endophytes and symbionts. For example, comparative genomic analyses of dark septate endophytes (DSEs) and *Trichoderma* spp. have shown that certain functional traits vary among lifestyles in fungi, BGC diversity often correlates more strongly with taxonomy than with environmental niche (Knapp et al. 2018; Scott et al. 2023). However, such studies remain limited for foliar fungi, especially in agricultural systems and in crops with edible leaves. Our work builds on this gap by evaluating the biosynthetic potential of phyllosphere-associated taxa in widely consumed leafy greens.

In parallel with BGCs, genomic features of such fungi may provide insight into biological roles that may differ among environments, including the potential to encode certain enzymes of important ecological function. For example, studies of root-associated fungi in arid systems, such as DSEs, have shown that fungi adapted to harsh, nutrient-poor soils often evolve expanded sets of carbohydrate-active enzymes (CAZymes), proteases, and melanin biosynthesis genes (Knapp et al. 2018). This suggests that functional enrichment of the genome may be a key adaptation to challenging environments. In arid environments specifically, fungi may face strong selective pressures that influence their secondary metabolism. Limited water availability, high UV radiation, temperature extremes, and low nutrient levels can drive the retention or expansion of BGCs involved in protective or competitive functions, such as melanin synthesis, antimicrobial compound production, or osmoprotectant pathways. Conversely, in stable, host-associated niches, certain BGCs may be lost or downregulated due to reduced ecological necessity, especially under domestication or reduced microbial competition (Knapp et al. 2018; Jing et al. 2022). However, leaf-associated fungi in similar arid settings may face very different selective pressures due to factors like exposure to ultraviolet (UV) radiation, temperature fluctuations, low water and nutrient availability, and antimicrobial compounds produced by plants (Leveau 2006; Arad 2025). In agricultural settings, these environmental pressures are further complicated by human manipulation, including breeding and pesticide application. Some leaf-associated fungi may lose or downregulate certain secondary metabolite pathways, especially under domestication or other human-mediated conditions. However, it remains unclear whether such streamlining is more strongly influenced by ecological filtering or by inherent genomic patterns associated with specific fungal lineages.

Given these environmental complexities, understanding how these dynamics play out in economically important crops like lettuce is especially relevant. Lettuce (*Lactuca sativa* L.), a globally grown crop, is a staple leafy vegetable in diets worldwide, providing vitamins, antioxidants, and minerals (Kim et al. 2016; Medina-Lozano et al. 2021). Because lettuce is frequently consumed raw, its leaf microbiota, including fungal communities, is of both ecological and health-related interest (Zhang et al. 2018b; Haelewaters et al. 2021; Damerum et al. 2021; Arad 2025). Lettuce also presents a useful model for testing hypotheses about ecological filtering due to its relatively simple leaf chemistry, short growing cycle, and common cultivation under arid, intensively managed conditions. Although bacterial communities on lettuce have received considerable attention (Williams et al. 2013; Williams and Marco 2014; Hunter et al. 2015; Luziatelli et al. 2019; Xu et al. 2024; Capparotto et al. 2024), fungal communities have not been studied as extensively, especially for non-pathogenic species. Previous studies show that lettuce leaves can host genera such as *Rhizoctonia*, *Sclerotinia*, *Bremia*, *Erysiphe*, *Olpidium*, and *Fusarium* (e.g., see acis.cals.arizona.edu). Some of these genera are known pathogens that cause diseases such as lettuce drop and downy mildew. In contrast, others may act as opportunists or exist more passively as part of the phyllosphere community. Previous studies have reported secondary metabolite BGCs from fungi isolated across various habitats (Franco et al. 2022; Liu et al. 2022; Ming et al. 2023; Scott et al. 2023; Villarreal Aguilar et al. 2024), but reports focusing on lettuce-associated fungi, particularly under arid agricultural conditions, remain scarce.

This study aims to characterize the biosynthetic potential of fungi isolated from the leaves of lettuce grown in arid agricultural conditions. We sequenced the genomes of nine representative isolates and predicted their secondary metabolite BGCs. To place our findings in a broader ecological and evolutionary context, we compared these findings with conspecific fungi from a range of habitats, using both isolates from our previous study and publicly available genomes. In this study, we define “lifestyle” as the biological association of the fungus with its host (e.g., epiphyte, endophyte, pathogen), whereas “ecological niche” refers more broadly to the environmental context, including host species, plant organ (e.g., leaf, root), and external abiotic conditions.

Leveraging this comparative framework, we hypothesized that lettuce-associated fungi may exhibit a reduced repertoire of BGCs, consistent with ecological filtering via domestication and human management of an edible leaf crop. Alternatively, phylogenetic constraints (i.e., species identity) could play the dominant role in shaping biosynthetic potential, and we will not observe a decrease in BGC content in the genomes of fungi simply on the basis of their occurring in lettuce leaves. To complement our analyses of secondary metabolism, we also examined CAZyme profiles in a species to assess whether host-associated differences in functional traits extend beyond BGCs, offering additional context for interpreting patterns of ecological filtering and phylogenetic constraint. Characterizing biosynthetic potential of lettuce-associated fungi can help identify both beneficial and potentially harmful compounds that affect crop quality and consumer health.

## Materials and methods

Lettuce leaves were collected in March 2023 from research plots at the Maricopa Agricultural Center (MAC) in Arizona, USA (33°04′24.8″ N, 111°58′25.7″ W). The site features Casa Grande sandy loam soil, with a mean annual temperature of 17.2 °C and average annual precipitation of 228.6 mm (Arad 2025). Three mature, healthy leaves were collected from representative individuals of five lettuce cultivars (Grand Rapids, Merlot, Iceberg, La Brillante, and Ninja) to obtain a wide sample of representative fungi. The field experiment is described in Arad (2025).

To isolate endophytic fungi, leaves were cut into ~ 2 mm^2^ pieces and surface-sterilized by agitation in 95% ethanol for 10 s, 0.5% NaOCl for 2 min, and then 70% ethanol for 2 min (Arnold et al. 2007). Segments were air-dried briefly in a laminar flow hood and plated onto potato dextrose agar (PDA) and malt extract agar (MEA). Plates were incubated at room temperature. For each plant, 10 leaf segments were randomly chosen for culture-based analysis. Any fungal colonies that emerged were transferred to fresh PDA or MEA to obtain pure isolates. For epiphytic fungi, fresh (non-sterilized) leaf segments (~ 2 mm^2^) were vortexed in 1 mL of sterile water. Then, a 100 µL portion of that wash was spread onto PDA and MEA plates using a sterile spreader. These plates also were incubated at room temperature. All culturing was performed using sterile technique in a laminar flow hood. Only sample plates showed fungal growth, and emerging colonies were morphologically consistent with phyllosphere fungi rather than common lab contaminants. All cultures are vouchered and maintained at the Robert L. Gilbertson Mycological Herbarium at the University of Arizona (Table 1). Isolates were identified tentatively by amplification and Sanger sequencing of the internal transcribed spacers and a portion of the adjacent nuclear ribosomal large subunit (ITS-partial LSUrDNA; see Arnold et al. 2007).

**Table 1:** Fungal strains isolated from leaves of arid field-grown lettuce

No	Isolate ID	Species	Medium	Lifestyle
1	AEH-2362	*Fulvia* sp. (*F. fulva*)	PDA	Epiphyte
2	AEH-2366	*Fusarium* sp. (*F. tjaetaba*)	PDA	Epiphyte
3	AEH-2368	*Alternaria alternata*	PDA	Epiphyte
4	AEH-2369	*F. tjaetaba*	PDA	Epiphyte
5	AEH-2430	*A. alternata*	PDA	Endophyte
6	AEH-2436	*Alternaria postmessia*	PDA	Endophyte
7	AEH-2437	*A. alternata*	MEA	Endophyte
8	AEH-2438	*A. postmessia*	MEA	Endophyte
9	AEH-2440	*A. alternata*	PDA	Endophyte

The table lists nine representative fungal isolates from arid field-grown lettuce leaves. Each strain represents a unique isolate, selected for taxonomic diversity and representing strains that were prevalent in our sampling of lettuce leaf-associated fungi

We selected nine isolates of lettuce leaf fungi for further analysis, representing epiphytic and endophytic lifestyles. These isolates were selected based on three criteria: (1) successful culturing, defined as consistent and sustained growth on PDA or MEA media over multiple subcultures; (2) taxonomic diversity, based on preliminary ITS-partial LSU sequencing that distinguished isolates at the genus or species level; and (3) frequency of occurrence, with priority given to taxa that were recovered from multiple plants and appeared across replicate culture plates. The isolates were identified based on ITS-partial LSUrDNA analyses as *Fusarium* sp., aligned with *Fusarium tjaetaba*; *Fulvia* sp., aligned with *Fulvia fulva*; *Alternaria alternata*; and *Alternaria postmessia*. Multiple strains were recovered each species (hereafter, *F. tjaetaba*, *A. alternata*, and *A. postmessia*) allowing for within-species comparisons (Table 1).

### Fungal DNA extraction and genome sequencing

Fungal mycelium was frozen at −80 °C, lyophilized, and pulverized using bead beating. Genomic DNA was extracted using the ZymoBIOMICS DNA Miniprep Kit (Zymo Research, Irvine, CA, USA). For library preparation, 25 ng of total DNA per sample was processed using the KAPA HyperPlus Kit (Roche, Basel, Switzerland) following a PCR-free workflow, with eight rounds of amplification to enhance library yield. Libraries were size-selected for 350–900 bp fragments using a combination of Zymo Select-a-Size Clean & Concentrator and gel purification. Final libraries were sequenced on an Illumina NovaSeq platform (Illumina, San Diego, CA, USA) with a target depth of ≥ 50 × genome coverage. The quality of the raw reads was assessed using FastQC v 0.12.1 (Andrews 2010). Raw reads were preprocessed with Trimmomatic v 0.39 (Bolger et al. 2014) to remove low-quality bases, trim adapters, and ensure that only high-quality sequences were retained for downstream analysis.

### Genome assembly and annotation

Genomes were assembled using SPAdes v 3.15.4 (Bankevich et al. 2012) with standard parameters, and annotations were carried out using BRAKER2 (Brůna et al. 2021). Basic genome statistics were calculated post-assembly to estimate genome size, GC content, and gene count (Table 2). To support species identification based on colony morphology and original DNA sequencing of the ITS-partial LSUrDNA, assembled sequences were compared against the National Center for Biotechnology Information (NCBI) fungal genome database (ncbi.nlm.nih.gov) using the basic local alignment search tool (BLAST) (blastn v 2.2.31 +), focusing on the identification of targeted fungal isolates.

**Table 2:** Summary of genome information used for comparative analysis in this study

No	NCBI genome assembly accession	Organism	Isolate/strain	Host	Host type	Source	Lifestyle	Collection site	Genome size (Mb)	GC content (%)	Number of genes
1	GCA_050710555.1	*Fulvia fulva*	AEH-2362	*Lactuca sativa*	Foliar lettuce	Leaf	Epiphyte	Arizona	33	53	7494
2	GCA_050711465.1	*Fusarium tjaetaba*	AEH-2366	*L. sativa*	Foliar lettuce	Leaf	Epiphyte	Arizona	54	49	16,121
3	GCA_050710795.1	*Alternaria alternata*	AEH-2368	*L. sativa*	Foliar lettuce	Leaf	Epiphyte	Arizona	35	51	13,056
4	GCA_050710735.1	*F. tjaetaba*	AEH-2369	*L. sativa*	Foliar lettuce	Leaf	Epiphyte	Arizona	50	49	15,243
5	GCA_050710675.1	*A. alternata*	AEH-2430	*L. sativa*	Foliar lettuce	Leaf	Endophyte	Arizona	35	51	13,019
6	GCA_050710595.1	*Alternaria postmessia*	AEH-2436	*L. sativa*	Foliar lettuce	Leaf	Endophyte	Arizona	38	51	13,191
7	GCA_050710565.1	*A. alternata*	AEH-2437	*L. sativa*	Foliar lettuce	Leaf	Endophyte	Arizona	36	51	13,460
8	GCA_050710805.1	*A. postmessia*	AEH-2438	*L. sativa*	Foliar lettuce	Leaf	Endophyte	Arizona	34	51	13,155
9	AEH-2440*	*A. alternata*	AEH-2440	*L. sativa*	Foliar lettuce	Leaf	Endophyte	Arizona	67	51	21,382
10	GCA_050711485.1	*A. alternata*	AEH-2221	*L. sativa*	Non-foliar lettuce	Root	Endophyte	Arizona	41	51	15,070
11	GCA_050711445.1	*A. postmessia*	AEH-2364B	*L. sativa*	Non-foliar lettuce	Soil	Epiphyte	Arizona	38	51	13,431
12	GCA_050710685.1	*A. postmessia*	AEH-2365	*L. sativa*	Non-foliar lettuce	Soil	Epiphyte	Arizona	35	52	13,053
13	GCA_049724655.1	*A. postmessia*	AEH-0273	*Lactuca serriola*	Non-foliar lettuce	Root	Endophyte	Arizona	35	51	13,488
14	GCA_049724695.1	*A. alternata*	AEH-3014	*L. serriola*	Non-foliar lettuce	Root	Endophyte	Arizona	34	52	13,137
15	GCA_049724635.1	*A. alternata*	AEH-3049	*L. serriola*	Non-foliar lettuce	Stem	Endophyte	Arizona	35	52	13,351
16	GCA_001572055.1	*A. alternata*	Z7	*Citrus suavissima*	Non-foliar lettuce	Soil	Pathogen	China	34	51	12,048
17	GCA_004154755.1	*A. alternata*	FERA 1177	*Malus domestica*	Non-foliar lettuce	Leaf	Pathogen	N/A	36	51	13,574
18	GCA_016097525.1	*A. alternata*	EV-MIL-31	*Citrus* x *tangelo*	Non-foliar lettuce	Leaf	Pathogen	Taiwan	35	51	24,515
19	GCA_020736535.1	*A. alternata*	MPI-PUGE-AT-0064	N/A	Non-foliar lettuce	N/A	Pathogen	N/A	33	51	13,192
20	GCA_034268455.1	*F. fulva*	2 4 9 11	*Solanum lycopersicum*	Non-foliar lettuce	Leaf	Pathogen	Poland	68	49	14,943
21	GCA_034269425.1	*F. fulva*	2 4 5 9 11 IPO	*S. lycopersicum*	Non-foliar lettuce	Leaf	Pathogen	Netherlands	67	49	14,944
22	GCA_035196885.1	*F. fulva*	Race 4	*S. lycopersicum*	Non-foliar lettuce	Leaf	Pathogen	Netherlands	67	49	14,895
23	GCA_035196915.1	*F. fulva*	0WU	*S. lycopersicum*	Non-foliar lettuce	Leaf	Pathogen	N/A	67	49	14,981
24	GCF_001642055.1	*A. alternata*	SRC1lrK2f	*Leucosceptrum canum*	Non-foliar lettuce	Leaf	Pathogen	China	33	52	13,572
25	GCF_013396195.1	*F. tjaetaba*	NRRL 66243	*Sorghum interjectum*	Non-foliar lettuce	Leaf	Pathogen	Australia	43	49	14,181
26	GCF_020509005.1	*F. fulva*	Race 5	*S. lycopersicum*	Non-foliar lettuce	Leaf	Pathogen	France	67	49	14,993
27	GCF_024291825.1	*A. postmessia*	BMP 2775	*Citrus* x *tangelo*	Non-foliar lettuce	Leaf	Pathogen	Florida	34	51	11,767

The table includes genome information for 27 fungal isolates: 9 from foliar lettuce-associated samples (this study) and 18 from other habitats (i.e., from previous collections by the authors (AEH) or from the NCBI database (GenBank assembly_ (GCA_)/RefSeq genome assembly_(GCF_)). The “other habitats” here means non-foliar lettuce-associated (abbreviated as Non-foliar lettuce). The “N/A” indicates that the information is not available. * The genome of isolate AEH-2440 is not currently deposited in NCBI but was included in the analyses. Its inclusion does not alter the overall results or conclusions

### antiSMASH and BiG-SCAPE analyses

Secondary metabolite BGCs were identified through analysis in antiSMASH v 7.1.0 via the web server, with default settings (Blin et al. 2023). To assess BGC similarity and group related clusters into gene cluster families (GCFs), we used the biosynthetic gene similarity clustering and prospecting engine (BiG-SCAPE) v 2.0.0b5 (Navarro-Muñoz et al. 2020) on the set of BGCs predicted by antiSMASH across all fungal genomes. All input files were GenBank-format region outputs (.gbk) generated directly from antiSMASH and no manual filtering was applied to the BGC dataset prior to analysis. BiG-SCAPE was executed in ‘cluster’ mode with the following parameters: “–input-mode flat,” “–gcf-cutoffs 0.3,” and “–mibig-v 4.0.” Protein domains were annotated using the Pfam database, which classifies protein families based on conserved domains. We specifically used the Pfam-A subset, which contains manually curated profile hidden Markov models (HMMs) for high-confidence domain assignments. The Pfam-A HMM database (Pfam-A.hmm) was obtained from Pfam release 33.1 (Mistry et al. 2021) and indexed using ‘hmmpress’ prior to analysis. Minimum information about a biosynthetic gene cluster (MIBiG) v 4.0 (Terlouw et al. 2023) was used to enable comparison with experimentally characterized BGCs.

Summary statistics for GCFs and BGC categories were calculated using a custom Python script. The scripts parsed BiG-SCAPE clustering output files (*_clustering_c0.3.tsv) to calculate the total number of BGCs processed, the number of unique GCFs identified, and the number of singletons versus clustered BGCs. They also computed the number of GCFs and total BGCs per biosynthetic class based on family assignments. Code generated for the present study is available at https://github.com/nedaarad25/Lettuce.

### CAZyme annotation

Predicted proteins were extracted using *GffRead*v 0.12.7 (Pertea and Pertea 2020) with the “-g” and “-y” flags, supplying genome sequences in FASTA format and capturing translated coding sequence (CDS) features in protein FASTA files. CAZyme annotation was conducted using the database for automated carbohydrate-active enzyme annotation (dbCAN3) meta server, which integrates three tools: (i) a hidden Markov model-based sequence alignment (HMMER) search against the dbCAN CAZyme domain HMM database for carbohydrate-active enzymes database (CAZy) family-level annotation, (ii) a double indexing alignment of next-generation sequencing data (DIAMOND) search against the CAZy for sequence similarity-based annotation, and (iii) a HMMER search against the dbCAN-sub HMM database, which provides CAZyme subfamily-level classification based on the enzyme class assignment by multi-level inference (eCAMI) clustering of the downloadable version of the CAZy (CAZyDB) families (Zhang et al. 2018a). Only annotations passing the default recommended thresholds were retained. Each tool was run with stringent cutoffs (HMMER: E-value < 1e − 15, coverage > 0.35; DIAMOND: E-value < 1e − 102), and only hits passing the recommended thresholds were retained. Gene models supported by at least two of the three tools were considered high-confidence CAZyme predictions. CAZy family assignments were extracted from the annotations for downstream analysis and comparative profiling.

To summarize CAZyme functional class distributions, we parsed family-level annotations from the dbCAN3 outputs using a custom Python script. Specifically, we extracted the alphabetic prefixes of CAZy domain labels using a regular expression that isolated the first contiguous uppercase string. These labels were used to classify each gene into broad CAZyme classes. Class frequencies were quantified using the ‘*value_counts()’* function in the ‘pandas’ library and the final results were summarized in a table and visualized as shown below. Code generated for the present study is available at https://github.com/nedaarad25/Lettuce.

### Comparative analysis

To enable comparative analysis, we included six genomes previously available in our laboratory collection (Table 2). We also downloaded publicly available genomes of the same species, isolated from different habitats, from NCBI genome database (Table 2). These were identified through a thorough search of the NCBI database for *F. tjaetaba*, *F. fulva*, *A. alternata*, and *A. postmessia*. Only genomes with accompanying GFF annotation files were included, as these are required by antiSMASH v 7.1.0 for BGC prediction.

We used JMP Pro (SAS Institute Inc., Cary, NC, USA; v 18.0.2) to perform a multiple linear regression (fit least squares platform), with BGC count as the response variable. Predictor variables included fungal species, host type (foliar vs. non-foliar), isolation source (leaf, root, stem, or soil), and lifestyle (epiphyte, endophyte, or pathogen), all treated as categorical factors. JMP applied dummy coding automatically. Model fit was evaluated with adjusted *R*^2^ values. Residual plots were visually inspected to evaluate assumptions of linearity and equal variance, and a normal quantile plot confirmed that residuals were approximately normally distributed.

## Results

Genome sizes for foliar lettuce-associated fungi ranged from 33 to 67 Mb, with GC content between 49 and 53% (Table 2). Their predicted gene counts varied widely across species and isolates, with *F. fulva* showing the lowest gene number (7,494), and one *A. alternata* isolate (AEH-2440) with over 21,000 genes (Table 2). Across all these foliar lettuce-associated fungal isolates, we identified 28 distinct BGC types (a total of 473 BGCs), including both canonical and hybrid cluster forms. These included clusters such as nonribosomal peptide synthetase (NRPS), type I polyketide synthase (T1PKS), terpene, fungal ribosomally synthesized and post-translationally modified peptide (RiPP)-like, and a range of hybrid types (NRPS + terpene, NRPS + T1PKS, and fungal-RiPP-like + NRPS) (Table 3).

**Table 3:** Distribution and frequency of BGCs in the foliar and non-foliar lettuce-associated fungal isolates

NCBI genome assembly accession	Betalactone	CDPS	Fungal-RiPP	Fungal-RiPP-like	Fungal-RiPP-like + isocyanide-NRP	Fungal-RiPP-like + NRPS	Fungal-RiPP + NRPS	Indole	Isocyanide	Isocyanide-NRP	Isocyanide-NRP + fungal-RiPP-like	Isocyanide-NRP + NRPS	Isocyanide + fungal-RiPP-like
GCA_050710555.1	1	0	0	3	0	1	1	0	0	1	0	0	0
GCA_050711465.1	1	1	0	3	0	0	0	2	1	0	0	0	0
GCA_050710795.1	0	0	1	12	0	0	0	0	0	1	0	0	0
GCA_050710735.1	1	1	0	6	0	0	0	2	1	0	0	1	0
GCA_050710675.1	0	0	1	12	0	0	0	0	0	1	0	0	0
GCA_050710595.1	0	0	1	11	0	0	0	0	0	1	0	0	0
GCA_050710565.1	0	0	1	10	0	0	0	0	0	1	0	0	0
GCA_050710805.1	0	0	1	12	0	0	0	0	0	1	0	0	0
AEH-2440*	1	0	2	14	0	1	0	1	1	1	0	0	0
GCA_050711485.1	0	0	1	12	0	0	0	0	0	1	0	0	0
GCA_050711445.1	0	0	1	8	0	1	0	0	0	1	0	0	0
GCA_050710685.1	0	0	1	9	0	0	0	0	0	1	0	0	0
GCA_049724655.1	0	0	1	10	0	0	0	0	0	0	1	0	0
GCA_049724695.1	0	0	1	7	1	0	0	0	0	0	0	0	0
GCA_049724635.1	0	0	1	10	0	0	0	0	0	1	0	0	0
GCA_001572055.1	0	0	1	12	0	0	0	0	0	1	0	0	0
GCA_004154755.1	0	0	1	10	0	0	0	0	0	1	0	0	0
GCA_016097525.1	1	0	1	10	0	0	0	0	1	1	0	0	0
GCA_020736535.1	0	0	1	11	0	0	0	0	1	1	0	0	0
GCA_034268455.1	0	0	2	14	0	0	0	0	0	0	0	0	1
GCA_034269425.1	0	0	2	14	0	0	0	0	0	0	0	0	1
GCA_035196885.1	0	0	2	15	0	0	0	0	1	0	0	0	0
GCA_035196915.1	0	0	2	15	0	0	0	0	1	0	0	0	0
GCF_001642055.1	0	0	1	10	0	0	0	0	0	1	0	0	0
GCF_013396195.1	1	1	0	3	0	1	0	1	2	0	0	1	0
GCF_020509005.1	0	0	2	16	0	0	0	0	1	0	0	0	0
GCF_024291825.1	1	0	1	10	0	0	0	0	0	1	0	0	0

NCBI genome assembly accession	NAPAA	NRP-metallophore + NRPS	NRP-metallophore + NRPS + fungal-RiPP-like	NRPS	NRPS-like	NRPS-like + NRPS	NRPS-like + T1PKS	NRPS + betalactone	NRPS + fungal-RiPP-like	NRPS + indole	NRPS + isocyanide-NRP	NRPS + NRPS-like	NRPS + T1PKS
GCA_050710555.1	1	1	0	6	11	0	1	0	0	0	0	0	0
GCA_050711465.1	0	1	0	8	9	0	0	0	0	0	1	0	3
GCA_050710795.1	1	1	0	4	6	0	0	0	0	0	0	0	2
GCA_050710735.1	0	1	0	7	9	0	0	0	0	0	0	1	2
GCA_050710675.1	1	1	0	4	6	0	0	0	0	0	0	0	0
GCA_050710595.1	1	1	0	3	6	0	0	0	0	0	0	0	0
GCA_050710565.1	1	1	0	2	6	0	0	0	0	0	0	0	0
GCA_050710805.1	1	1	0	3	6	0	0	0	0	0	0	0	2
AEH-2440*	1	1	0	14	15	0	0	0	0	2	0	0	1
GCA_050711485.1	1	1	0	3	6	0	1	0	0	0	0	0	0
GCA_050711445.1	1	1	0	6	7	0	0	0	0	0	0	0	1
GCA_050710685.1	1	1	0	3	6	0	0	0	1	0	0	1	2
GCA_049724655.1	1	1	0	3	7	0	1	0	0	0	0	0	0
GCA_049724695.1	0	1	0	2	2	0	1	0	0	0	0	0	1
GCA_049724635.1	1	1	0	3	6	0	0	0	0	0	0	0	1
GCA_001572055.1	1	1	0	3	6	0	0	0	0	0	0	0	3
GCA_004154755.1	1	1	0	3	8	0	0	0	0	0	0	1	2
GCA_016097525.1	1	0	0	4	7	0	0	0	0	0	0	0	2
GCA_020736535.1	1	1	0	3	6	0	0	0	0	0	0	0	1
GCA_034268455.1	1	0	1	7	12	0	0	1	1	0	0	0	0
GCA_034269425.1	1	0	1	6	12	0	0	1	1	0	0	0	0
GCA_035196885.1	1	0	1	6	12	0	0	1	1	0	0	0	0
GCA_035196915.1	1	0	1	6	12	0	0	1	1	0	0	0	0
GCF_001642055.1	1	1	0	4	6	0	0	0	0	0	0	0	2
GCF_013396195.1	0	1	0	7	7	1	0	0	0	1	0	0	1
GCF_020509005.1	1	0	1	8	12	0	0	1	1	0	0	0	1
GCF_024291825.1	1	1	0	5	6	0	0	0	0	0	0	0	1

NCBI genome assembly accession	NRPS + T1PKS + indole	NRPS + terpene	Phosphonate	T1PKS	T1PKS + fungal-RiPP-like	T1PKS + NRPS	T1PKS + NRPS-like	T1PKS + terpene	T3PKS	Terpene	Terpene + NRPS-like	Terpene + T1PKS	Total
GCA_050710555.1	0	0	0	7	1	0	0	0	1	7	0	0	43
GCA_050711465.1	1	0	0	16	0	2	0	0	1	11	0	0	61
GCA_050710795.1	0	0	0	8	0	1	0	0	1	7	0	0	45
GCA_050710735.1	1	0	0	14	0	4	0	1	1	12	0	0	65
GCA_050710675.1	0	0	0	8	0	2	0	0	1	7	0	0	44
GCA_050710595.1	0	0	0	7	0	1	0	0	1	6	0	0	39
GCA_050710565.1	0	0	0	7	0	2	0	0	1	7	0	0	39
GCA_050710805.1	0	0	0	8	0	0	0	0	1	6	0	0	42
AEH-2440*	0	1	0	21	0	4	0	0	1	12	1	0	95
GCA_050711485.1	0	0	0	8	0	1	0	0	1	6	0	0	42
GCA_050711445.1	0	0	0	9	0	2	0	0	1	7	0	0	46
GCA_050710685.1	0	0	0	9	0	1	0	0	1	8	0	0	45
GCA_049724655.1	0	0	0	10	0	1	0	0	1	6	0	0	43
GCA_049724695.1	0	0	0	2	0	0	0	0	1	6	0	0	25
GCA_049724635.1	0	0	0	8	0	0	0	0	1	7	0	0	40
GCA_001572055.1	0	0	0	7	0	1	0	0	1	6	0	0	43
GCA_004154755.1	0	0	0	8	0	0	0	0	0	6	0	0	42
GCA_016097525.1	0	0	0	8	0	0	1	0	1	6	0	0	44
GCA_020736535.1	0	0	0	7	0	2	0	0	1	6	0	0	42
GCA_034268455.1	0	0	0	6	0	4	1	0	0	3	0	0	54
GCA_034269425.1	0	0	0	6	0	4	1	0	0	3	0	0	53
GCA_035196885.1	0	0	0	6	0	4	1	0	0	3	0	0	54
GCA_035196915.1	0	0	0	6	0	4	1	0	0	4	0	0	55
GCF_001642055.1	0	0	0	9	0	0	0	0	1	7	0	0	43
GCF_013396195.1	1	0	1	10	0	3	1	0	1	10	0	1	56
GCF_020509005.1	0	0	0	7	0	2	1	0	0	4	0	0	58
GCF_024291825.1	0	0	0	7	0	0	0	0	1	5	0	0	40

The table summarizes BGC counts by class across 27 fungal genomes: 9 isolated from leaves of lettuce and 18 from other habitats. BGCs were predicted independently for each genome using antiSMASH. Each value represents the total count of BGCs across all genomes in that group. CDPS = Cyclodipeptide synthase; RiPP = Ribosomally synthesized and post-translationally modified peptide; RiPP-like = RiPP cluster with incomplete or divergent biosynthetic features compared to characterized RiPPs; NRP = Nonribosomal peptide; NRPS = Nonribosomal peptide synthetase; NRPS-like = NRPS-related biosynthetic gene cluster with partial domain architecture; NAPAA = Non-α-poly-amino acid; RiPP = Ribosomally synthesized and post-translationally modified peptide; T1PKS = Type I polyketide synthase; T3PKS = Type III polyketide synthase. Cluster combinations (e.g., NRPS + T1PKS) reflect hybrid biosynthetic gene clusters containing features of multiple classes. * The genome of isolate AEH-2440 is not currently deposited in NCBI but was included in the analyses. Its inclusion does not alter the overall results or conclusions

The total number of BGCs per foliar lettuce-associated isolate ranged from 39 to 95, with notable variation among strains of the same species (Table 3). *A. alternata* strain AEH-2440 exhibited the highest BGC count, with 95 clusters, including a wide range of canonical and hybrid types. In contrast, *A. postmessia* strain AEH-2436 and *A. alternata* strain AEH-2437 carried the lowest total BGC counts, each with 39 clusters. Most isolates showed complex BGC profiles, with multiple hybrid combinations (Table 3).

**Fig. 1:**
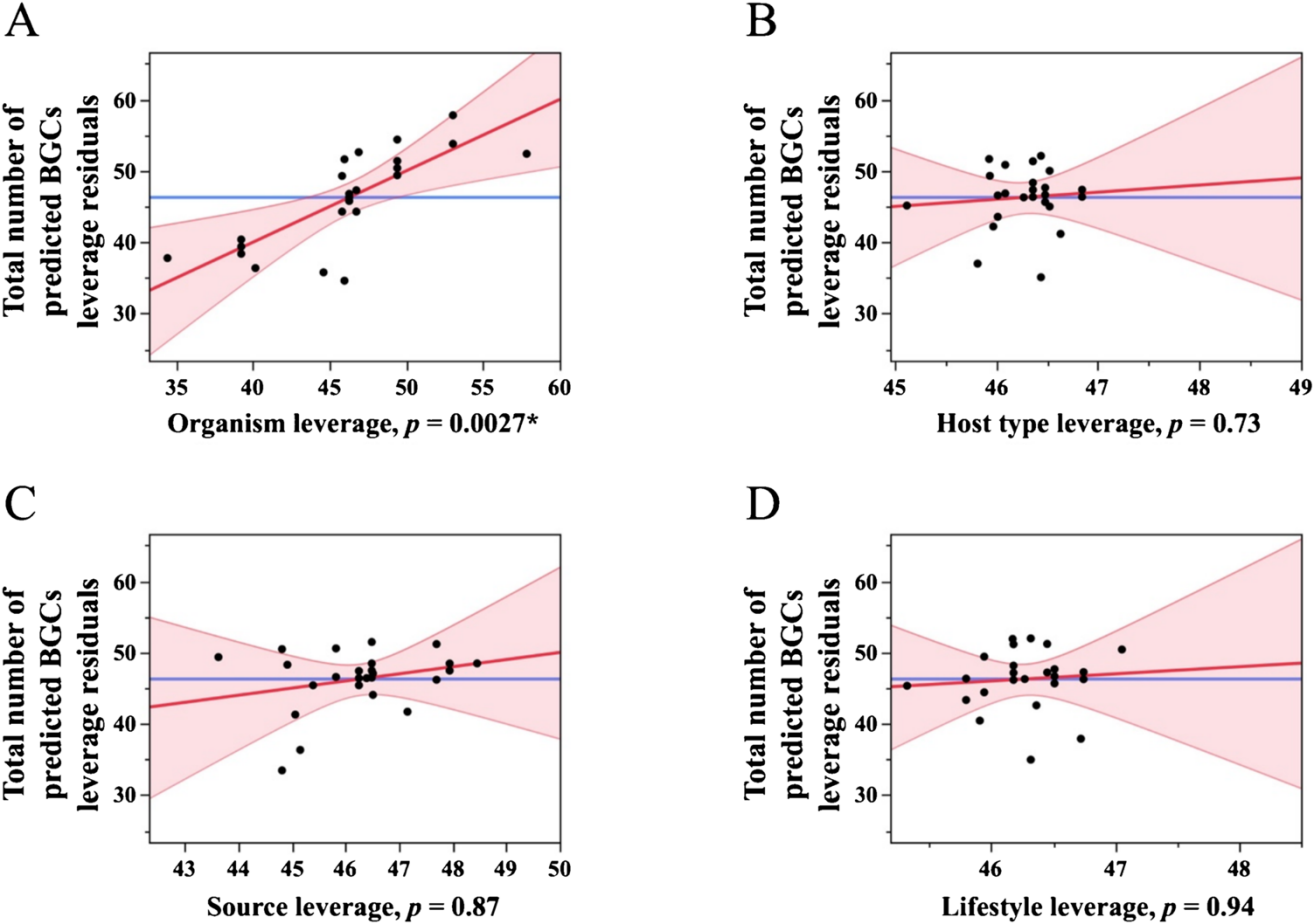
Regression analysis of BGC counts across fungal isolates, including foliar and non-foliar lettuce-associated origins. Regression analysis of BGC counts across 27 fungal isolates from foliar and non-foliar lettuce-associated leaves substrates. Each point represents a single genome. The linear regression model includes categorical predictors (organism (fungal species), host type, source, and lifestyle; see main text for details). Panels represent leverage plots that show the impact of each factor on the model as a whole, with the line of fit colored by confidence curves in each case

Among non-foliar lettuce-associated fungal isolates, a total of 825 BGCs were identified across 18 genomes, with individual isolates containing between 25 and 58 clusters (Table 3). These included a wide range of BGC types, such as fungal-RiPP-like, NRPS, T1PKS, and terpenes, along with numerous hybrid combinations (NRPS + T1PKS and fungal-RiPP-like + NRPS). Similar to the foliar lettuce-associated fungi, fungal-RiPP-like clusters were again among the most frequently observed. Notably, *F. fulva* race 5 exhibited the highest BGC count among non-foliar lettuce-associated strains, with a total of 58 clusters (Table 3). Fungal species was a significant predictor of BGC number (*p* = 0.0027), while host type (foliar lettuce vs. non-foliar lettuce), isolation source (e.g., leaf vs. root), and lifestyle (e.g., endophyte vs. epiphyte) were not (*p* = 0.73, 0.87, and 0.94, respectively; Fig. 1; Tables 4, 5, 6).

**Table 4:** Leverage and least squares means by group

**Leverage**	**Level**	**Least sq mean**	**Std error**	**Mean**	
**Organism**	*Alternaria alternata*	39.94	3.49	40.81	
	*Alternaria postmessia*	40.87	5.23	42.50	
	*Fulvia fulva*	50.12	4.57	52.83	
	*Fusarium tjaetaba*	58.56	4.64	60.66	
**Host type**	Foliar lettuce-associated	45.98	7.52	47.25	
	Non-foliar lettuce-associated	48.76	2.20	45.83	
**Source**	Leaf	49.57	1.75	48.72	
	N/A	48.71	5.79	42.00	
	Root	42.61	8.06	36.66	
	Soil	49.71	4.96	44.66	
	Stem	46.25	9.61	40.00	
**Lifestyle**	Endophyte	47.15	3.27	39.25	
	Epiphyte	48.27	3.36	50.83	
	Pathogen	46.69	8.50	48.66	

Estimated marginal means (least squares means) and standard errors from a linear regression model predicting BGC count. Values are based on n = 27 genomes and reflect group-level estimates adjusted for all other variables in the model. Leverage indicates the influence of each factor level on fitted values. Least Sq = Least squares mean; Std error = Standard error. The “N/A” indicates that the information is not available

**Table 5:** Summary of analysis of variance (ANOVA) and model fit statistics for the linear regression model predicting BGC counts

Analysis of variance					
**Source**	**DF**		**Sum of squares**	**Mean square**	***F* ratio**
Model	10		1403.58	140.35	5.21
Error	15		403.52	26.90	Prob > *F*
Total	25		1807.11		0.0022*
**Lack of fit**					
**Source**	**DF**		**Sum of squares**	**Mean square**	***F* ratio**
Lack of fit	4		216.72	54.18	3.19
Pure error	11		186.80	16.98	Prob > *F*
Total error	15		403.52		0.057
					**Max RSq**
					0.89
**Summary of fit**					
RSquare		0.77			
RSquare adj		0.62			
Root mean square error		5.18			
Mean of response		46.26			
Observations (or sum wgts)		26			

Summary statistics from the ANOVA table associated with the regression model of BGC count. Based on data from n = 27 fungal genomes. Model fit was evaluated using adjusted R^2^ and residual diagnostics. DF = Degrees of freedom; RSquare = Coefficient of determination; RSquare adj = Adjusted RSquare; Root mean square error = Standard deviation of the residuals; Prob > *F* = *p*-value associated with the *F*-test; Max RSq = Maximum possible RSquare under the model; Observations (or sum wgts) = Total number of data points or sum of weights used in the model

**Table 6:** Parameter estimates for the regression model predicting BGC count

Term	Estimate	Std error	*t* ratio	Prob >|*t*|
Intercept	47.37	3.80	12.45	<.0001*
Organism [*Alternaria alternata*]	−7.43	1.89	−3.92	0.0014*
Organism [*Alternaria postmessia*]	−6.50	2.66	−2.44	0.027*
Organism [*Fulvia fulva*]	2.74	2.20	1.25	0.23
Host type [foliar lettuce]	−1.39	4.03	−0.34	0.73
Source [leaf]	2.19	4.86	0.45	0.65
Source [N/A]	1.34	6.04	0.22	0.82
Source [root]	−4.76	5.23	−0.91	0.37
Source [soil]	2.34	3.87	0.60	0.55
Lifestyle [endophyte]	−0.21	4.10	−0.05	0.95
Lifestyle [epiphyte]	0.89	2.67	0.33	0.74

Parameter estimates, standard errors, *t*-values, and *p*-values from the multiple regression model predicting BGC count (n = 27 fungal genomes). Asterisks indicate predictors significant at *p* < 0.05. The “N/A” indicates that the information is not available

### Analyses of focal BGCs

To further illustrate the biosynthetic potential present in our analyzed genomes, we selected two known BGCs with relevance to human health and ecological functions for closer inspection: alternariol and 1,3,6,8-Tetrahydroxynaphthalene (T4HN). Alternariol is a well-characterized mycotoxin with known genotoxic and estrogenic effects in mammals, but in fungi, it may also serve roles in microbial competition and host tissue colonization (Lehmann et al. 2006; Wenderoth et al. 2019). T4HN is an intermediate in the melanin biosynthesis pathway, which contributes to fungal resistance against UV radiation, oxidative stress, and desiccation  traits particularly advantageous in the arid phyllosphere (Zhang et al. 2003; Tanaka et al. 2015).

**Fig. 2:**
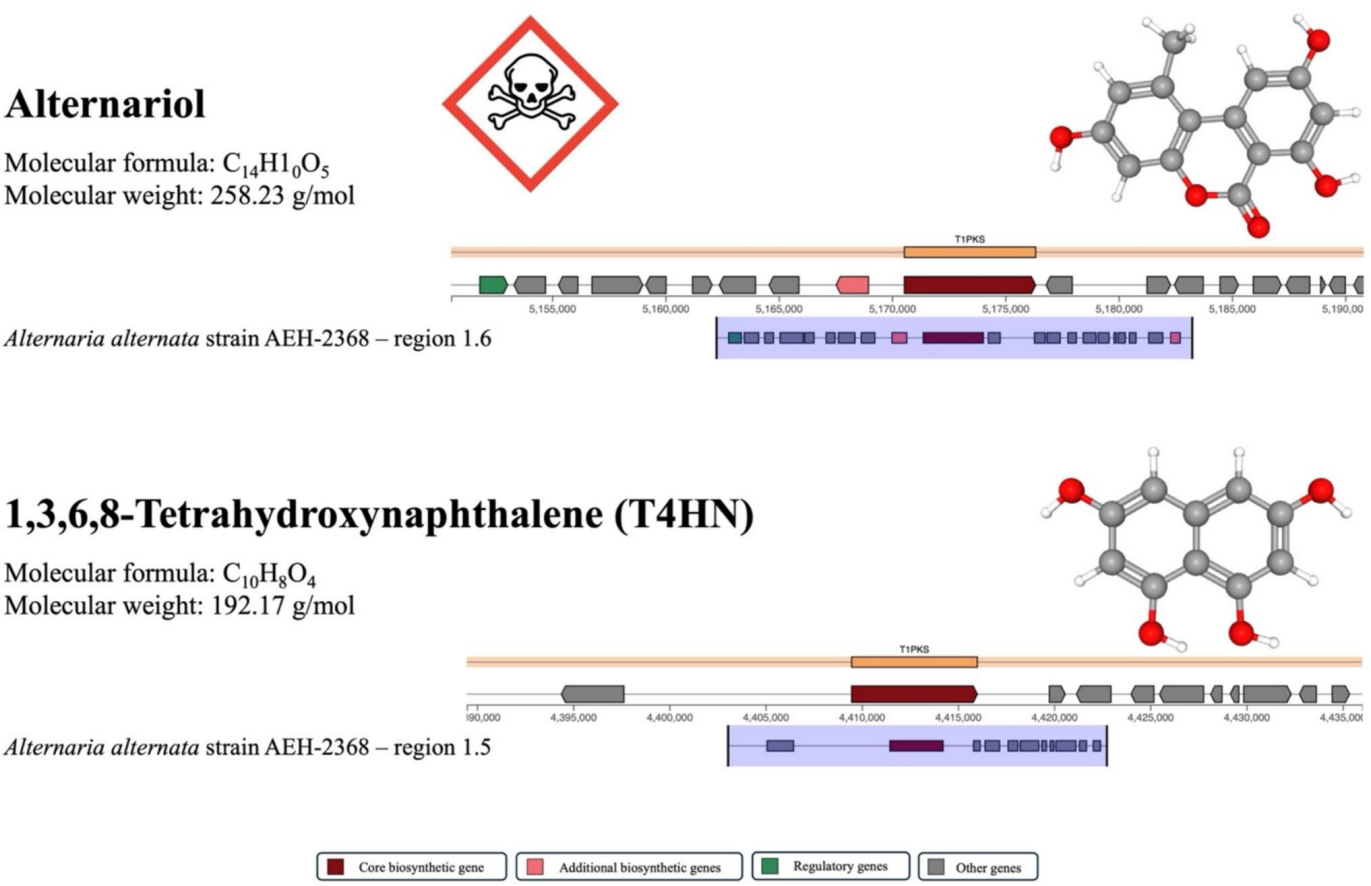
Biosynthetic gene cluster organization and chemical structures of alternariol and 1,3,6,8-Tetrahydroxynaphthalene (T4HN). Schematic representations of the BGCs for alternariol (top) and T4HN (bottom) identified in *Alternaria alternata* strain AEH-2368. Gene cluster visualizations generated using antiSMASH v 7.1.0 web server and modified for clarity. Gene functions are color-coded: core biosynthetic (dark red), additional biosynthetic (pink), transport-related (blue), regulatory (green), and other genes (grey). The corresponding molecular structures and properties are obtained from the NCBI pubchem database (pubchem.ncbi.nlm.nih.gov) and shown alongside each BGC. Gene cluster annotations are based on similarity to reference clusters BGC0001284 (alternariol) and BGC0001258 (T4HN) from the MIBiG database. Each “region” corresponds to a specific BGC assigned by antiSMASH within the genome

The alternariol cluster, classified as a polyketide BGC, was detected in 16 isolates, including 10 *A. alternata* and 6 *A. postmessia* strains (Table 7). It showed high similarity to the reference cluster BGC0001284 in the MIBiG database (mibig.secondarymetabolites.org). This cluster has been experimentally validated in *A. alternata* previously and is known to encode the biosynthesis of alternariol (Chooi et al. 2015) (Fig. 2).

**Table 7:** Distribution of known BGCs across the genomes analyzed in this study

NCBI genome assembly accession	Region	Most similar known cluster	BGC class	Similarity
GCA_050710555.1	Region 1.1	Clavaric acid	Terpene	100%
GCA_050710555.1	Region 1.3	Choline	NRP	100%
GCA_050710555.1	Region 23.1	1,3,6,8-Tetrahydroxynaphthalene	Polyketide	100%
GCA_050711465.1	Region 2.1	Choline	NRP	100%
GCA_050711465.1	Region 45.1	Bikaverin	Polyketide	100%
GCA_050711465.1	Region 59.2	Alternapyrone	Polyketide	100%
GCA_050711465.1	Region 61.1	Koraiol	Terpene	100%
GCA_050711465.1	Region 82.1	Depudecin	Polyketide:Iterative type I polyketide	100%
GCA_050710795.1	Region 1.1	Alternapyrone	Polyketide	100%
GCA_050710795.1	Region 1.5	1,3,6,8-Tetrahydroxynaphthalene	Polyketide	100%
GCA_050710795.1	Region 1.6	Alternariol	Polyketide	100%
GCA_050710795.1	Region 7.1	Choline	NRP	100%
GCA_050710735.1	Region 14.2	Choline	NRP	100%
GCA_050710735.1	Region 22.1	Oxyjavanicin	Polyketide	100%
GCA_050710735.1	Region 32.2	ACT-toxin II	Polyketide	100%
GCA_050710735.1	Region 39.1	α-Acorenol	Terpene	100%
GCA_050710735.1	Region 51.2	Bikaverin	Polyketide	100%
GCA_050710735.1	Region 56.2	Alternapyrone	Polyketide	100%
GCA_050710735.1	Region 80.1	Choline	NRP	100%
GCA_050710735.1	Region 106.1	Koraiol	Terpene	100%
GCA_050710735.1	Region 124.1	Fusatrixin/fusapentaxin/fusaoctaxin A	NRP	100%
GCA_050710675.1	Region 1.2	Alternariol	Polyketide	100%
GCA_050710675.1	Region 1.3	1,3,6,8-Tetrahydroxynaphthalene	Polyketide	100%
GCA_050710675.1	Region 10.1	Choline	NRP	100%
GCA_050710675.1	Region 18.1	Alternapyrone	Polyketide	100%
GCA_050710595.1	Region 3.2	1,3,6,8-Tetrahydroxynaphthalene	Polyketide	100%
GCA_050710595.1	Region 3.3	Alternariol	Polyketide	100%
GCA_050710595.1	Region 4.2	Alternapyrone	Polyketide	100%
GCA_050710595.1	Region 10.1	Choline	NRP	100%
GCA_050710565.1	Region 1.4	1,3,6,8-Tetrahydroxynaphthalene	Polyketide	100%
GCA_050710565.1	Region 1.5	Alternariol	Polyketide	100%
GCA_050710565.1	Region 2.1	Alternapyrone	Polyketide	100%
GCA_050710565.1	Region 12.1	Choline	NRP	100%
GCA_050710565.1	Region 21.1	1-Heptadecene	Polyketide:modular type I polyketide	100%
GCA_050710805.1	Region 1.2	1,3,6,8-Tetrahydroxynaphthalene	Polyketide	100%
GCA_050710805.1	Region 1.3	Alternariol	Polyketide	100%
GCA_050710805.1	Region 2.1	Alternapyrone	Polyketide	100%
GCA_050710805.1	Region 5.1	Choline	NRP	100%
AEH-2440*	Region 2.1	Alternariol	Polyketide	100%
AEH-2440*	Region 38.1	1,3,6,8-Tetrahydroxynaphthalene	Polyketide	100%
AEH-2440*	Region 50.1	Choline	NRP	100%
AEH-2440*	Region 77.1	Clavaric acid	Terpene	100%
AEH-2440*	Region 200.1	Alternapyrone	Polyketide	100%
AEH-2440*	Region 251.1	Clavaric acid	Terpene	100%
AEH-2440*	Region 619.1	Choline	NRP	100%
GCA_050711485.1	Region 1.1	Alternapyrone	Polyketide	100%
GCA_050711485.1	Region 1.5	1,3,6,8-Tetrahydroxynaphthalene	Polyketide	100%
GCA_050711485.1	Region 1.6	Alternariol	Polyketide	100%
GCA_050711485.1	Region 19.1	Choline	NRP	100%
GCA_050711445.1	Region 1.2	1,3,6,8-Tetrahydroxynaphthalene	Polyketide	100%
GCA_050711445.1	Region 1.3	Alternariol	Polyketide	100%
GCA_050711445.1	Region 25.1	Choline	NRP	100%
GCA_050711445.1	Region 29.1	Alternapyrone	Polyketide	100%
GCA_050711445.1	Region 80.1	Mellein	Polyketide	100%
GCA_050710685.1	Region 1.2	1,3,6,8-Tetrahydroxynaphthalene	Polyketide	100%
GCA_050710685.1	Region 1.3	Alternariol	Polyketide	100%
GCA_050710685.1	Region 18.1	Alternapyrone	Polyketide	100%
GCA_050710685.1	Region 23.1	Choline	NRP	100%
GCA_050710685.1	Region 86.1	Mellein	Polyketide	100%
GCA_049724655.1	Region 1.4	1,3,6,8-Tetrahydroxynaphthalene	Polyketide	100%
GCA_049724655.1	Region 3.1	Choline	NRP	100%
GCA_049724655.1	Region 18.1	Alternariol	Polyketide	100%
GCA_049724655.1	Region 28.1	Alternapyrone	Polyketide	100%
GCA_049724655.1	Region 30.2	Mellein	Polyketide	100%
GCA_049724635.1	Region 1.1	Alternapyrone	Polyketide	100%
GCA_049724635.1	Region 4.1	Choline	NRP	100%
GCA_049724635.1	Region 8.1	Alternariol	Polyketide	100%
GCA_049724635.1	Region 24.1	1,3,6,8-Tetrahydroxynaphthalene	Polyketide	100%
GCA_001572055.1	Region 10.1	Alternariol	Polyketide	100%
GCA_001572055.1	Region 21.1	Choline	NRP	100%
GCA_001572055.1	Region 35.1	Mellein	Polyketide	100%
GCA_001572055.1	Region 48.1	ACT-toxin II	Polyketide	100%
GCA_001572055.1	Region 58.1	Mellein	Polyketide	100%
GCA_004154755.1	Region 1.1	Choline	NRP	100%
GCA_004154755.1	Region 1.4	Alternariol	Polyketide	100%
GCA_004154755.1	Region 68.1	Alternapyrone	Polyketide	100%
GCA_004154755.1	Region 83.1	Asperlactone	Polyketide	100%
GCA_004154755.1	Region 499.1	AM-toxin	NRP	100%
GCA_016097525.1	Region 1.2	Alternariol	Polyketide	100%
GCA_016097525.1	Region 1.3	1,3,6,8-Tetrahydroxynaphthalene	Polyketide	100%
GCA_016097525.1	Region 10.2	Mellein	Polyketide	100%
GCA_016097525.1	Region 17.1	Mellein	Polyketide	100%
GCA_016097525.1	Region 31.1	ACT-toxin II	Polyketide	100%
GCA_016097525.1	Region 34.2	Alternapyrone	Polyketide	100%
GCA_016097525.1	Region 61.1	Choline	NRP	100%
GCA_020736535.1	Region 1.2	Alternapyrone	Polyketide	100%
GCA_020736535.1	Region 3.1	Choline	NRP	100%
GCA_020736535.1	Region 20.3	Alternariol	Polyketide	100%
GCA_034268455.1	Region 8.1	UNII-YC2Q1O94PT	Polyketide	100%
GCA_034268455.1	Region 13.3	Choline	NRP	100%
GCA_034269425.1	Region 8.1	UNII-YC2Q1O94PT	Polyketide	100%
GCA_034269425.1	Region 13.3	Choline	NRP	100%
GCA_035196885.1	Region 8.1	UNII-YC2Q1O94PT	Polyketide	100%
GCA_035196885.1	Region 13.3	Choline	NRP	100%
GCA_035196915.1	Region 8.1	UNII-YC2Q1O94PT	Polyketide	100%
GCA_035196915.1	Region 13.3	Choline	NRP	100%
GCF_001642055.1	Region 3.1	1,3,6,8-Tetrahydroxynaphthalene	Polyketide	100%
GCF_001642055.1	Region 7.1	Choline	NRP	100%
GCF_001642055.1	Region 24.1	Alternapyrone	Polyketide	100%
GCF_013396195.1	Region 49.1	Choline	NRP	100%
GCF_013396195.1	Region 76.1	α-Acorenol	Terpene	100%
GCF_013396195.1	Region 146.1	Peramine	NRP	100%
GCF_013396195.1	Region 368.1	Monascorubrin	Polyketide	100%
GCF_013396195.1	Region 379.1	Koraiol	Terpene	100%
GCF_020509005.1	Region 8.1	UNII-YC2Q1O94PT	Polyketide	100%
GCF_020509005.1	Region 13.3	Choline	NRP	100%
GCF_024291825.1	Region 113.1	Alternariol	Polyketide	100%
GCF_024291825.1	Region 122.1	ACT-toxin II	Polyketide	100%
GCF_024291825.1	Region 168.1	Choline	NRP	100%
GCF_024291825.1	Region 301.1	Alternapyrone	Polyketide	100%

BGCs were predicted using antiSMASH for each of the 27 genomes and matched against the MIBiG database. Only exact matches (100% similarity) to known clusters are shown. Each entry represents a BGC identified in a specific genome. “Region” refers to the numerical identifier assigned to each BGC by antiSMASH within a given genome. NRP = Nonribosomal peptide; UNII = Unique Ingredient Identifier assigned by the U.S. Food and Drug Administration (FDA) Substance Registration System; ACT = *Alternaria citri* toxin. * The genome of isolate AEH-2440 is not currently deposited in NCBI but was included in the analyses. Its inclusion does not alter the overall results or conclusions

The T4HN cluster, categorized under the polyketide class, was identified in 14 isolates, including 8 *A. alternata,* 5 *A. postmessia*, and 1 *F. fulva* (Table 7). The T4HN biosynthetic gene cluster found in our dataset displayed significant similarity to the reference cluster BGC0001258 in the MIBiG database. This cluster has been experimentally confirmed in *Glarea lozoyensis* in previous studies and is recognized for encoding T4HN biosynthesis (Zhang et al. 2003) (Fig. 2).

### Potential relationships of BGCs at the domain level

Of the 1,298 BGCs predicted by antiSMASH across the 27 fungal genomes, 1,146 were retained and processed by BiG-SCAPE for clustering analysis. These BGCs included 354 NRPS, 285 RiPP, 232 PKS, and 158 terpene clusters, along with 84 NRPS + PKS hybrids, 12 NRPS + RiPP hybrids, 11 classified as other, 5 NRPS + other hybrids, 3 NRPS + PKS + other hybrids, and 2 RiPP + other hybrids. Clustering resulted in the identification of 175 GCFs, with all BGCs assigned to a family and no singletons observed. The number of GCFs detected per class included 54 NRPS, 32 RiPP, 36 PKS, 26 terpene, 17 NRPS + PKS, 3 NRPS + RiPP, 4 other, 1 NRPS + other, 1 NRPS + PKS + other, and 1 RiPP + other.

### CAZyme analyses in a focal species

**Fig. 3:**
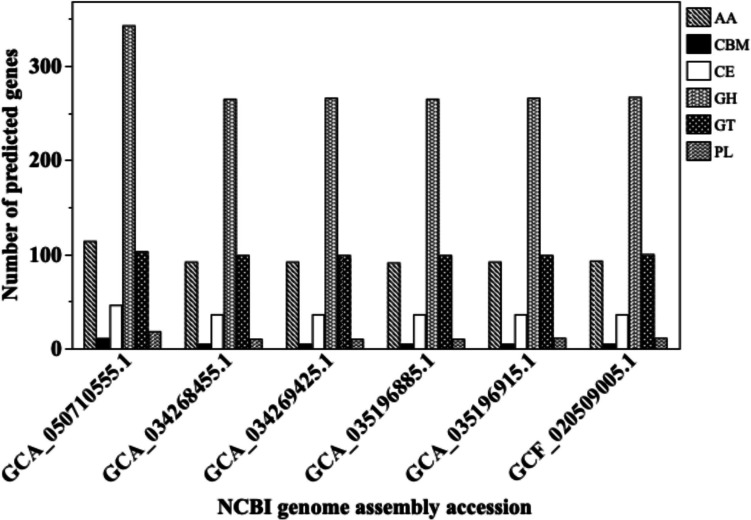
Comparison of CAZyme class abundance in a *Fulviafulva* isolate from lettuce compared with strains from tomato. Bar plots show the number of predicted genes assigned to major CAZyme classes across six *F. fulva* genomes. One isolate (AEH-2362 (GCA_050710555.1)) was recovered from lettuce, while the remaining five were isolated from tomato (obtained from publicly available genome assemblies in NCBI). Gene predictions were made using the dbCAN2 pipeline. Each bar represents a single genome; no technical replicates are included. CAZyme classes include glycoside hydrolases (GH), glycosyltransferases (GT), auxiliary activities (AA), carbohydrate esterases (CE), polysaccharide lyases (PL), and carbohydrate-binding modules (CBM)

To further explore functional profiles beyond secondary metabolism, we analyzed CAZymes in *F. fulva*. This species was selected due to its comparatively large genome and gene count in our dataset, suggesting a higher potential for functional diversity. Moreover, *F. fulva* was isolated from both lettuce and tomato (*Solanum lycopersicum*), two economically important dicotyledonous vegetables that, despite their taxonomic distance, share ecologically similar leaf environments characterized by human-influenced selective pressures. A total of 1,946 CAZyme-related genes were identified in *F. fulva* isolated from foliar lettuce (AEH-2362), spanning several functional classes. The majority belonged to the glycoside hydrolases (GH, 342 genes), followed by auxiliary activities (AA, 113), glycosyltransferases (GT, 102), carbohydrate esterases (CE, 45), polysaccharide lyases (PL, 17), carbohydrate-binding modules (CBM, 11), and unclassified (N, 1,316). Across *F. fulva* isolates from tomato, GHs were again the most abundant class (ranging from 264 to 266 genes), followed by GTs (98 and 99), AAs (90–92), CEs (35), PLs (9 and 10), CBMs (5), and Ns (1,187–1,191). Total CAZyme counts were generally lower in the tomato isolates (1688–1698) (Fig. 3).

## Discussion

This study offers one of the first genome-level perspectives on the secondary metabolite potential of leaf-associated fungi from arid field-grown lettuce. By examining genome data for representative epiphytic and endophytic fungi, we assessed how their predicted BGC profiles compare against conspecific strains from other habitats. We initially hypothesized that foliar lettuce-associated fungi would show reduced BGC diversity due to ecological filtering or domestication, but our findings suggest a more nuanced picture. BGC counts varied widely across isolates from both foliar and non-foliar lettuce-associated, and regression analysis indicated that species identity, rather than host type, source, or lifestyle, was a significant predictor of BGC richness. This highlights the importance of phylogenetic context when interpreting functional genomic traits in plant-associated fungi.

More broadly, this work builds on the growing body of research showing that endophytic and epiphytic fungi play important roles in plant resilience and ecological dynamics, often via the production of specialized metabolites encoded by biosynthetic gene clusters (Rodriguez et al. 2009; Fadiji and Babalola 2020a; Liu et al. 2022). Although the present study did not validate gene expression nor measure it in planta nor in the field, our study points to BGCs of interest for biosynthetic potential. Previous studies show clearly that BGC content can vary even among closely related fungi depending on habitat (Knapp et al. 2018; Jing et al. 2022), but our results suggest that such variation may be more strongly tied to species identity, and we could not detect strong patterns within species according to lifestyle or other factors. Earlier studies have proposed that domestication and host-driven ecological filtering might lead to genome streamlining in plant-associated microbes (Knapp et al. 2018; Rokas et al. 2020; Jing et al. 2022), but our data suggest that species identity exerts a stronger influence on biosynthetic potential than host environment or niche. For example, *A. alternata* strains showed a wide range of BGC counts, yet regression analysis indicated that lifestyle (e.g., epiphyte vs. endophyte) was not a significant predictor. Although factors such as exposure to abiotic stress in the phyllosphere (Leveau 2006) or niche-specific plant interactions (Hardoim et al. 2015; Arad 2025) may influence fungal behavior or metabolite expression, we did not find systematic differences in genome structure as a function of such factors among the fungi considered here.

Several factors may contribute to the observed strain-level variation in BGC content within fungal species. One possibility is horizontal gene transfer, which has been documented in fungi and may introduce novel BGCs into specific lineages (Slot and Rokas 2011; Gardiner et al. 2012). Additionally, the presence or absence of certain BGCs may be influenced by mobile genetic elements, such as transposons, that mediate gene gain or loss events (Ma et al. 2010). Differences in genome assembly quality or completeness could also influence BGC predictions, although we attempted to minimize this by using only genomes with available GFF annotations and ≥ 50 × sequencing depth (Yadav and Subramanian 2024). Finally, strain-specific ecological interactions, such as competitive dynamics within the phyllosphere or prior adaptation to other hosts, may shape the retention or loss of BGCs, even among genetically similar isolates (Trivedi et al. 2020; Mukherjee et al. 2023). Further research can explore each of these, highlighting a potential benefit of the existing culture collection from the present study.

Notably, some biosynthetic capacities seem to be conserved across foliar lettuce-associated fungi, especially for compounds with dual ecological and health relevance. The polyketide BGCs for alternariol and T4HN were detected in 16 and 14 isolates, respectively, spanning multiple *A. alternata* and *A. postmessia* strains, as well as one *F. fulva* isolate. T4HN plays a role in melanin production and stress tolerance (Knapp et al. 2018), and alternariol is a well-known mycotoxin with documented genotoxic and estrogenic effects in humans (Chooi et al. 2015). Since lettuce leaves are often eaten raw, the presence of fungi capable of producing such compounds, even at low frequency or under specific conditions, is of note (Zhang et al. 2018b; Damerum et al. 2021). The fact that these clusters are retained even in isolates with otherwise low BGC counts suggests they may confer critical survival or colonization advantages or at the very least, are not strongly selected against in this niche. However, determining whether these genes are actively expressed and whether the corresponding metabolites are produced in planta or during post-harvest stages will be the next step in evaluating their ecological roles and food safety implications, and in distinguishing between gene loss versus regulatory control as drivers of reduced biosynthetic potential.

Previous work has suggested that domesticated crops like lettuce might select for functionally streamlined microbial partners. Our results do not support a consistent reduction in biosynthetic gene content among foliar lettuce-associated fungi. Instead, BGC richness varied widely across isolates, regardless of host source or lifestyle. That said, the relatively short life cycle and chemically simple leaf environment of lettuce (Leveau 2006; Arad 2025) may still influence the expression or ecological relevance of certain secondary metabolites. Rather than broad gene loss, the impact of domestication and host traits may play out more subtly, through shifts in regulatory dynamics or selective retention of key biosynthetic pathways.

Our previous study showed that fungal richness in lettuce leaves is already relatively low (Arad 2025), consistent with some degree of ecological filtering. However, our findings suggest that this filtering may not result in consistent reductions in biosynthetic gene content. Instead, functional impacts may be more nuanced, affecting which clusters are expressed, retained, or activated under specific conditions. Repeated human interventions such as pesticide use and selective breeding may further shape fungal communities and their traits (Medina-Lozano et al. 2021), making lettuce, and perhaps other fast-growing leafy vegetables, a particularly interesting model for exploring how microbes cope with short-lived, human-managed environments. In contrast, longer-lived crops with more chemically complex foliage may support fungi with broader or more diverse BGC repertoires (Villarreal Aguilar et al. 2024).

Multiple BGCs identified in our dataset, including those for alternariol and T4HN, showed 100% similarity to their respective entries in the MIBiG database, based on antiSMASH comparisons. While BiG-SCAPE identified numerous GCFs, some BGCs with high sequence similarity to known MIBiG clusters were not consistently grouped, likely due to differences in clustering methodology between antiSMASH and BiG-SCAPE. Comparisons by antiSMASH are based on nucleotide-level identity and annotated boundaries of known clusters, whereas BiG-SCAPE groups clusters based on Pfam domain content, order, and synteny (Navarro-Muñoz et al. 2020). As a result, even highly conserved clusters can be placed in separate GCFs or remain ungrouped if minor differences in domain annotation or arrangement are present, a well-documented limitation of domain-based clustering in fungi (Robey et al. 2021; Jo et al. 2023). This challenge is especially pronounced in fungal genomes, where variation in domain prediction, gene order, or assembly quality can affect clustering outcomes (Robey et al. 2021; Jo et al. 2023). Similar patterns have been reported in other studies of fungal endophytes and symbionts, where BGCs often remain unclustered despite high sequence similarity (Knapp et al. 2018; Rokas et al. 2020). These results support the use of complementary approaches, such as both antiSMASH and BiG-SCAPE, to more fully capture and interpret BGC diversity in fungal genomes. It is also important to acknowledge the limitations of these prediction tools. antiSMASH relies on predefined gene models and domain annotations, which can lead to missed or misclassified clusters in fragmented or lower-quality assemblies (Robey et al. 2021; Blin et al. 2021). Similarly, BiG-SCAPE’s domain-based clustering is sensitive to gene order, synteny, and annotation differences, which can obscure true biological similarity (Navarro-Muñoz et al. 2020). As such, predicted BGC counts and groupings should be interpreted as approximations rather than exhaustive inventories, and complementary validation approaches may be needed to fully characterize functional potential.

To contextualize our findings on BGC repertoire, we also analyzed CAZyme profiles in *F. fulva* isolates from lettuce and tomato. Although CAZymes are not directly linked to secondary metabolism, their abundance reflects broader genomic capacity for plant interaction and environmental adaptation, providing a complementary perspective on how host niche may shape fungal genomic traits (Zhao et al. 2013). The lettuce isolate carried 1,946 CAZyme-related genes, including elevated counts in glycoside hydrolases (342) and auxiliary activity enzymes (102). In contrast, tomato-derived *F. fulva* isolates showed lower total CAZyme counts (1,688–1,698), and lower gene counts in several key classes, including glycoside hydrolases (264–266), auxiliary activities (90–92), and glycosyltransferases (98–99). These enzymes are important for plant cell wall modification and nutrient access. Tomato is often described as having chemically complex and defensive traits in foliage (Zhang et al. 2003), whereas lettuce is generally considered to offer a more chemically mild and stable habitat (Kim et al. 2016; Medina-Lozano et al. 2021). These results suggest that host chemical complexity alone may not fully explain fungal genomic capacity.

One limitation of this study is the absence of biological replicates for the sequenced fungal genomes. Our goal was to capture a broad range of taxa and host associations, which limited our ability to include technical replicates. As such, we cannot fully assess how technical variation or subtle within-strain genomic differences might influence BGC prediction outcomes. Future work incorporating replicated sequencing or expression-based validation could help refine our understanding of variability in biosynthetic potential across and within fungal taxa. Additionally, it is important to note that all BGC predictions presented here are computational and represent potential biosynthetic capacity rather than direct evidence of metabolite expression or activity. Experimental approaches such as transcriptomics, proteomics, or metabolite profiling would be required to confirm the functional expression and ecological roles of the predicted clusters (e.g., see Jia et al. 2016; Kautsar et al. 2019; Navarro-Muñoz et al. 2020).

Understanding the biosynthetic potential of phyllosphere fungi has implications for microbiome engineering, sustainable agriculture, and food safety. Microbiome research has largely focused on bacterial taxa, but fungi play critical roles in shaping plant health, nutrient cycling, and pathogen resistance (Backer et al. 2018). Our findings contribute genomic insight into culturable lettuce-associated fungi, some of which may produce bioactive metabolites relevant for crop protection or growth promotion (Gupta et al. 2024). As interest grows in designing microbial consortia to reduce chemical inputs or enhance stress tolerance, genome-informed selection of fungal isolates could support development of targeted biostimulants or biocontrol agents for lettuce production. Additionally, recognizing the phylogenetic constraints on biosynthetic capacity may help predict the functional potential of fungal members within future microbiome assemblies or synthetic communities.

This study provides a genome-level view of biosynthetic and functional diversity in fungi associated with lettuce, a widely consumed leafy crop. Our findings challenge assumptions about ecological filtering and lifestyle-based streamlining. While certain clusters, like those for alternariol and T4HN, appear conserved across multiple isolates, overall biosynthetic capacity varies even within species, independent of host source or fungal niche. CAZyme comparisons further suggest that host identity may influence functional potential, but not always in ways predicted by host chemistry alone. Together, these results highlight the complexity of plant–microbe interactions in managed agroecosystems and reinforce the importance of broader comparative sampling, especially in raw-consumed crops where microbial metabolism may intersect with food safety, quality, and resilience.

## Data Availability

Sequence data from the genomes isolated in this study are available at NCBI GenBank under BioProject accession number PRJNA1264501.
